# Differences in responses to English and Korean versions of the Caregiver Priorities & Child Health Index of Life with Disabilities (CPCHILD)

**DOI:** 10.1186/s12955-020-01528-4

**Published:** 2020-08-17

**Authors:** Ki Hyuk Sung, Soon-Sun Kwon, Gyeong Hee Cho, Chin Youb Chung, Clarissa Encisa, Huroy Menal, Unni G. Narayanan, Moon Seok Park

**Affiliations:** 1 Department of Orthopaedic Surgery, Seoul National University Bundang Hospital, 82 Gumi-ro, 173 Beon-gil, Bundang-gu, Sungnam, Gyeonggi 13620 South Korea; 2 Department of Mathematics, College of Natural Sciences, Ajou University, Gyeonggi, South Korea; 3 Division of Orthopaedics & Child Health Evaluative Sciences Program, The Hospital for Sick Children, University of Toronto, 555 University Avenue, S-107, Toronto, Ontario M5G 1X8 Canada

**Keywords:** Cerebral palsy, Questionnaire, Social environment, Korean, English

## Abstract

**Background:**

The purpose of this study was to identify differences in caregiver responses to Korean-language and English-language versions of the Caregiver Priorities & Child Health Index of Life with Disabilities (CPCHILD) questionnaire.

**Methods:**

Patient data were acquired from the Cerebral Palsy Hip Outcomes Project database, which was established to run a large international multicenter prospective cohort study of the outcomes of hip interventions in cerebral palsy. Thirty-three children whose caregivers had completed the Korean version of CPCHILD were matched by propensity scoring with 33 children whose parents completed the English version. Matching was performed on the basis of 12 covariates: age, gender, gross motor function classification system level, migration percentage of right and hip, seizure status, feeding method, tracheostomy status, pelvic obliquity, spinal deformity, parental report of hip pain and contracture interfering with care.

**Results:**

There were no significant differences in CPCHILD scores for section 4 (Communication and Social Interaction), and section 5 (Health) between two groups. Korean-language CPCHILD scores were significantly lower than English-language CPCHILD scores for section 1 (Personal Care/Activities of Daily Living), section 2 (Positioning, Transferring and Mobility), section 3 (Comfort and Emotions) and section 6 (Overall Quality of Life) as well as in terms of total score.

**Conclusions:**

Cultural influences, and the community or social environment may impact the caregivers’ perception of the health-related quality of life of their children. Therefore, physicians should consider these differences when interpreting the study outcomes across different countries.

## Background

Cerebral palsy (CP) comprises a group of permanent disorders of movement and posture that are attributed to a non-progressive disturbance in the developing fetal or infant brain [1]. Reduced activity levels and participation restrictions due to these disorders may lead to a reduced quality of life (QOL) in children with CP compared to typically developing peers [2, 3]. Due to an increasing emphasis placed on measuring health-related QOL (HRQOL) in patients with CP, various assessment tools and questionnaire have been developed. The Caregiver Priorities & Child Health Index of Life with Disabilities (CPCHILD) was developed to use caregivers’ perspectives to assess the HRQOL of children with severe developmental disabilities [4]. The CPCHILD has been reported as one of the most valid measures of QOL in children with CP [5].

The CPCHILD questionnaire has been translated and adapted in several languages and cultures. The Dutch, German, Scandinavian, and Korean versions of the CPCHILD have shown relevant validity in terms of known-group validity, convergent validity, internal consistency, and test-retest reliability [6–9]. Recently, the CPCHILD questionnaire was used to evaluate QOL-related outcomes after hip reconstructive surgery among children with CP, and previous studies have used the CPCHILD questionnaire to report improved HRQOL after surgery [10, 11].

International standards guide the process of transcultural adaptation and validation of questionnaires between different languages [12–14], and the CPCHILD questionnaire has been translated, adapted, and validated according to these rules. Although the CPCHILD has been validated according to international guidelines, there is a concern when comparing the scores of original questionnaires and translations. Translation and transcultural adaptation attempt to render similar meanings between two languages but do not always reflect cultural, community-related or social differences, which can impact responses to the questions. For example, would parent respondents of children who are otherwise similar, generate similar scores in Korean and English-speaking communities when the culture and society of each community is potentially vastly different?

The purpose of this study was to explore the assumption that patients with similar demographic and comorbid profiles would generate similar CPCHILD scores in different countries. Specifically, this study aimed to identify differences in responses between versions of the CPCHILD by comparing scores between matched participants in Korea and several English-language countries.

## Methods

This study was approved by the institutional review board of our hospital, which is a tertiary referral center for patients with CP. The need to acquire additional consent was waived because of the secondary nature of this study. Patient data were acquired from the existing Cerebral Palsy Hip Outcomes Project (CHOP) database [15], which was established to run a large multicenter prospective cohort study of the outcomes of hip interventions in cerebral palsy being conducted at 28 sites in 11 countries. There were 41 Korean-speaking and 406 English-speaking participants in the CHOP database at the time of this study (January 2018).

### CPCHID questionnaire

The CPCHILD questionnaire consists of 37 items over six domains: Personal Care/Activities of Daily Living (Section 1, 9 items); Positioning, Transferring and Mobility (Section 2, 8 items); Comfort and Emotions (Section 3, 9 items); Communication and Social Interaction (Section 4, 7 items); Health (Section 5, 3 items); and Overall QOL (Section 6, 1 item) [4, 7]. For sections 1, 2 and 4, each item was rated on a 7-point ordinal scale from 0 to 6. For sections 3, 5 and 6, each item was rated on a 6-point ordinal scale from 0 to 5. Standardized scores from 0 (worst) to 100 (best) were calculated for each of the six domains as well as the total survey. These scores were derived from the raw item scores divided by the maximum item score, multiplied by 100. A higher score corresponds to a higher QOL.

### Propensity score matching

Differences in characteristics between the Korean-speaking group and English-speaking group were adjusted using propensity score matching to reduce selection bias. Propensity scores represent the relationship between the multiple characteristics and the status of each case. In brief, the score is the probability of receiving a case status. The single score was calculated using multivariate logistic regression with the SAS Proc LOGISTIC procedure. A greedy algorithm was used to match the Korean-speaking group and the English-speaking group. The Korean-speaking group was first matched to the English-speaking group on five digits of the propensity score. For those that did not match, four-digit matches were identified, and this process continued down to a one-digit match of propensity scores [16].

In this study, propensity score matching was performed on the basis of 12 covariates: age, migration percentages of the right and left hip, gender, Gross Motor Function Classification System (GMFCS) level, seizure status, feeding method, tracheostomy status, pelvic obliquity, spinal deformity, parent report of hip pain and contracture interfering with care. Two groups were formed using 1:1 matching and their differences in baseline CPCHILD scores were analyzed.

### Statistical analysis

Descriptive statistics including the mean, standard deviation, and proportion were used to summarize patient demographics. The Shapiro-Wilk test verified the normality of the distribution of variables. Differences in patient demographics and CPCHILD scores between the two groups were analyzed using the chi-square test and two-tailed t-test. Data were analyzed using SAS version 9.4.2 (SAS Institute Inc., Cary, NC, USA). All tests were two-tailed, and *p*-values < 0.05 were considered statistically significant.

## Results

Two groups were established on the basis of propensity score matching from the current CHOP cohort. One group comprised 33 patients whose caregivers completed the Korean version of the CPCHILD (mean age 8.8 ± 3.4 years, 22 males and 11 females) and the other group comprised 33 patients whose caregivers completed the English version of CPCHILD (mean age 8.0 ± 3.5 years, 23 males and 10 females). Korean-speaking participants were from South Korea, and English-speaking participants were from Canada (*n* = 1), the United States (*n* = 23), Australia (*n* = 3), New Zealand (*n* = 1) and the United Kingdom (*n* = 5).

No significant differences between groups were observed in demographic variables, including age, migration percentages of the right and left hip, sex, GMFCS level, involvement (unilateral vs. bilateral), seizure status, feeding method, tracheostomy status, history of fragility fracture, history of spine surgery, history of non-hip related surgical procedure, use of intrathecal baclofen pump, spinal deformity, and parental report of hip pain or contracture interfering with care. However, there was significant difference in pelvic obliquity between groups (Table 1). There were no significant differences in caregiver demographics, including age, gender, working status, relationship to child, and education level between groups (Table 2).

**Table 1:** Patients demographics

	Korean version (*N* = 33)	English version (*N* = 33)	*p*-value
Age (year)	8.8 ± 3.4	8.5 ± 3.4	0.758
Gender (Male / Female)	22 / 11	24 / 9	0.789
GMFCS level (IV / V)	14 / 19	15 / 18	1.000
Distribution of involvement (unilateral / bilateral)	0 / 33	0 / 33	1.000
MP (right hip, %)	59.6 ± 28.0	50.2 ± 26.5	0.058
MP (left hip, %)	58.1 ± 26.1	52.6 ± 32.5	0.371
Seizure status (No seizure / Controlled / Poorly controlled)	14 / 17 / 2	16 / 15 / 2	1.000
Feeding method (Oral only / Oral and G-tube / G-tube only)	29 / 1 / 3	28 /2 / 3	0.879
Tracheostomy status (Yes / No)	1 / 32	0 / 33	1.000
History of fragility fracture (Yes / No)	1 / 32	0 / 33	1.000
History of spine surgery (Yes / No)	1 / 32	0 / 33	1.000
Intrathecal baclofen pump (Yes / No)	0 / 33	5 / 28	1.000
History of non-hip related surgical procedure (Yes / No)	1 / 32	9 / 24	0.063
Pelvic obliquity (Yes / No)	8 / 25	14 / 19	0.016
Spinal deformity (Yes / No)	12 / 21	11 / 22	1.000
Parent report of Hip Pain (Yes / No)	13 / 30	13 / 20	1.000
Parent report of Contracture interfering with care (Yes / No)	25 / 8	23 / 10	0.782

*GMFCS* Gross Motor Functional Classification System, *MP* migration percentage

**Table 2:** Caregivers demographics

	Korean version	English version	*p*-value
Age (year)	42.8 ± 6.3	40.8 ± 8.2	0.273
Gender (Male / Female)	6 / 27	5 / 28	0.741
Working status (not working / working full or part time)	23 / 10	16 / 14	0.205
Relationship to child (biological parent / adoptive parent / professional caregiver / other)	29 / 0 / 3 / 1	27 / 3 / 0 / 0	0.058
Education level (some high school or less / high school diploma / vocational school or some college / college or university degree / professional or graduate degree)	1 / 10 / 9 / 9 / 4	2 / 3 / 6 / 12 / 4	0.329

There were no significant differences in CPCHILD scores for section 4 (Communication and Social Interaction; *p* = 0.182), and section 5 (Health; *p* = 0.547) between the two groups. Korean parents reported significantly lower CPCHILD scores than English-speaking parents in section 1 (Personal Care and Activities of Daily Living; *p* = 0.026), section 2 (Positioning, Transferring, and Mobility; *p* = 0.001), section 3 (Comfort and Emotions; *p* = 0.018), and section 6 (Overall QOL; *p* < 0.001) and in terms of the total score (*p* = 0.007). The mean total score for the English CPCHILD (54.3 ± 11.7) responses was 1.3 times higher than that for the Korean CPCHILD (42.6 ± 19.4) responses (Table 3). The largest differences in the CPCHILD mean scores between the two groups were in section 6 (21.2; 95% CI, 9.6 to 32.8) followed by section 3 (16.4; 95% CI, 4.1 to 28.7) and section 2 (13.2; 95% CI, 3.7 to 22.8).

**Table 3:** Comparison of CPCHILD scores between Korean and English language-base populations

Section	Content	Korean version (*N* = 33)	English version (*N* = 33)	*p*-value
1st section	Personal care/activities of daily living	28.1 ± 21.4	38.2 ± 16.7	0.026
2nd section	Positioning, transferring and mobility	24.8 ± 21.0	38.0 ± 16.8	0.001
3rd section	Comfort and emotions	59.9 ± 28.8	76.3 ± 19.4	0.018
4th section	Communication and social interaction	48.1 ± 28.4	58.4 ± 23.0	0.182
5th section	Health	69.4 ± 20.2	67.4 ± 17.4	0.547
6th section	Overall quality of life	46.7 ± 27.2	67.9 ± 18.0	< 0.001
Total		42.6 ± 19.4	54.3 ± 11.7	0.007

*CPCHILD* Caregiver Priorities & Child Health Index of Life with Disabilities

## Discussion

In children who are otherwise similar, parents from English speaking countries report significantly higher CPCHILD total scores than the Korean parents, as well as higher scores in the subscales for Personal Care and Activities of Daily living; Positioning, Transferring and Mobility; Comfort and Emotion; and Overall QOL. Similar scores were found between the two groups in the subscales of Communication and Social Interaction, and Health.

Several factors may explain the differences between the English-language and Korean-language CPCHILD responses. Language-related factors intrinsic to the questionnaires used, such as the subtle nuances of meaning or understanding that cannot be directly translated, could influence the responses. However, the translation and transcultural adaptations are performed according to the international guidelines, which are believed to correspond to the best available current practice. During the transcultural adaptation process, the equivalence between the source and target version is evaluated in four areas, that is, semantic, idiomatic, experiential, and conceptual equivalence [12]. Semantic equivalence indicates whether the words mean the same thing. Regarding idiomatic equivalence, colloquialisms or idioms are difficult to translate. Thus, the committee has to formulate an equivalent expression in the target version. In terms of experiential equivalence, a given task may simply not be experienced in a different country or culture even if it is translatable. Therefore, the questionnaire item would have to be replaced by a similar item that is in fact experienced in the target culture. Conceptual equivalence indicates whether the conceptual meaning between cultures is different. It is notable that the transcultural adaptation process does not consider the community and social background.

Aside from linguistic factors, caregiver responses to QOL questionnaires could also be influenced by the community or social environment, and differences between these environments in Korean and English-speaking contexts could have influenced differences in the responses in our study. For example, differences were noted in positioning, transferring, and mobility between the two groups. These differences could be influenced by the presence or absence of available public infrastructure to facilitate accessibility, such as ramps for wheelchairs, which could have influenced the caregivers’ responses to difficulty in “moving about outdoors” and “visiting public places”. For these items in section 2, scores in the English CPCHILD were significantly higher than those in the Korean CPCHILD. Cultural differences and societal attitudes could have influenced the difference in responses for items in the subscales of section 3, 5, and 6. In section 3 responses, reports of pain or discomfort “while eating/drinking or being fed,” “during toileting,” “while dressing/undressing,” “during transfers or position changes” and “while seated” were significantly lower in the Korean CPCHILD than in the English CPCHILD. In section 5 and 6, scores regarding the child’s overall health and overall QOL were also significantly lower in the Korean CPCHILD than in the English CPCHILD (Table 4).

**Table 4:** Comparison of CPCHILD scores for each item between Korean and English language-base populations

Section	Item	Korean version (N = 33)	English version (*N* = 33)	*p*-value
1st section	1	4.3 ± 2.5	4.5 ± 2.4	0.753
	2	3.5 ± 2.39	3.1 ± 1.8	0.432
	3	2 ± 2.0	2.2 ± 1.9	0.565
	4	2.8 ± 2.6	2.7 ± 2.2	0.922
	5	2.1 ± 2.2	2.4 ± 1.8	0.568
	6	2.1 ± 2.1	2.9 ± 2.0	0.127
	7	2.1 ± 2.2	2.2 ± 1.6	0.666
	8	2.1 ± 2.3	2.3 ± 1.8	0.593
	9	2.1 ± 2.3	3.4 ± 2.3	0.024
2nd section	10	3 ± 2.7	2.5 ± 2.0	0.713
	11	2.1 ± 2.3	2.4 ± 1.7	0.369
	12	3.1 ± 2.7	5.3 ± 2.9	0.004
	13	1.4 ± 2.1	1.7 ± 1.8	0.216
	14	3.4 ± 2.8	3.7 ± 2.8	0.732
	15	1.7 ± 2.5	3.2 ± 2.7	0.012
	16	1.5 ± 2.2	1.8 ± 1.6	0.129
	17	1.5 ± 2.2	2.8 ± 1.5	< 0.001
3rd section	18	4.1 ± 2.7	6.1 ± 1.6	0.001
	19	3.1 ± 2.7	5.7 ± 1.9	< 0.001
	20	3.0 ± 2.7	5.7 ± 2.0	< 0.001
	21	3.2 ± 2.6	5.5 ± 1.8	< 0.001
	22	3.6 ± 2.7	5.7 ± 2.0	< 0.001
	23	4.5 ± 2.8	5.4 ± 1.9	0.313
	24	4.4 ± 2.9	5.4 ± 1.8	0.339
	25	4.6 ± 2.1	5.2 ± 1.7	0.292
	26	5.3 ± 2.2	5.4 ± 1.5	0.607
4th section	27	2.8 ± 2.3	3.9 ± 1.8	0.081
	28	3.5 ± 2.0	3.6 ± 2.0	0.934
	29	2.5 ± 1.9	2.0 ± 2.0	0.416
	30	2.8 ± 2.1	2.9 ± 2.3	0.816
	31	2.8 ± 2.0	2.8 ± 2.1	0.958
	32	3 ± 2.1	4.2 ± 1.6	0.029
	33	2.3 ± 2.0	3.6 ± 1.8	0.012
5th section	34	3.7 ± 1.6	4.1 ± 1.1	0.488
	35	2.8 ± 1.1	3.6 ± 1.0	0.003
	36	3.6 ± 1.7	2.3 ± 1.8	0.005
6th section	37	2.2 ± 1.4	3.4 ± 0.8	< 0.001

*CPCHILD* Caregiver Priorities & Child Health Index of Life with Disabilities

The impact of activity restrictions and limitation of participation in persons with disabilities are of international interest. Disability arises from a mismatch between individual ability and environmental needs [17]. QOL in individuals with disabilities is influenced not only by the functional abilities but also by the environment which can pose both as a barrier or a facilitator. Governmental laws and regulations, such as the American Disability Act (ADA) in the US and similar regulations in other English-speaking countries, mandate enforceable standards to ensure accessibility for public transportation, and public settings and services. These policies reduce the impact of activity restrictions imposed by the severity of the CP. The items of the CPCHILD span all domains of the International Classification of Functioning, Disability and Health including environmental factors, and has the potential to assess the efficacy of government policy and enforcement of regulations. However, this needs to be validated in a future study.

This study has some limitations. First, although the groups were closely matched for the selected covariates, there remains the possibility that the groups might differ substantially in other areas that influence the CPCHILD scores. Potentially confounding factors such as individual caregivers’ socio-economic status and education level were not controlled for between the two groups. There was no significant difference in caregiver education level between groups. However, we could not acquire information regarding the caregivers’ socio-economic status. Second, English-speaking responses included participants from a variety of countries, such as Canada, US, Australia, and Great Britain. Responses were not compared between each English-language country, as more participants than our available sample size would be required for such a comparison. The US has mixed health care system that includes public or private funding for the care of children with disabilities, while the three other English-speaking countries all have national or provincial wide publicly funded health care systems. Nevertheless, these countries are comparable in socio-economic terms.

## Conclusions

In conclusion, this study revealed significant differences in caregiver responses between English-language and Korean-language versions of the CPCHILD, even though they were otherwise similar in characteristics that might influence their HRQOL. Cultural influences, environmental factors, and community or social contexts may impact caregivers’ perception of QOL of their children. Therefore, physicians should consider these differences when interpreting the study outcomes across different countries.

## Data Availability

The data set supporting the conclusion of this article is available on request to the corresponding author.

## Abbreviations

CP: cerebral palsy
QOL: quality of life
CPCHILD: The Caregiver Priorities & Child Health Index of Life with Disabilities
GMFCS: Gross Motor Function Classification System
